# Association of sleep patterns and water intake with cognitive functions in adults in an urban environment

**DOI:** 10.12669/pjms.40.4.8268

**Published:** 2024

**Authors:** Shazia Qayyum, Samina Iltaf, Madiha Sajjad, Mashaal Azam

**Affiliations:** 1 Shazia Qayyum, PhD, Department of Pathology, Riphah International University, Rawalpindi, Pakistan; 2 Samina Iltaf, MPhil, Department of Pathology, Riphah International University, Rawalpindi, Pakistan; 3 Madiha Sajjad, FCPS, Department of Pathology, Riphah International University, Rawalpindi, Pakistan; 4 Mashaal Azam, MBBS. House Officer Surgery Dept. Pakistan Railway Hospital, RIU, Islamabad, Pakistan

**Keywords:** Cognitive function, Cognitive performance, Mood, Sleep patterns, Water intake, Water supplementation, Working adults

## Abstract

**Objectives:**

To explore the behavior patterns of students and working adults regarding the duration and quality of sleep and water intake in an urban environment and to identify the relationship between nighttime sleep extent and water intake with mood and cognitive performance.

**Methods:**

This was a descriptive correlational study conducted at Islamic International Medical College, Riphah University at the Pathology department from March to June 2022. A total of 160 participants with age range of 20 to 50 years completed a self-report questionnaire regarding sleep patterns, water intake, and perceptions of mood, concentration, and memory. Analyzed the relationship between sleep duration, water intake, mood, mindfulness/concentration, and memory using Kendall’s Tau-b correlation coefficient in SPSS 22.

**Results:**

A significant number (28.7%) of participants had a sleep duration of ≤ 6 hours, with 41.3% sleeping after midnight. 82.5% of the participants switch off-screen just before sleeping. 63.7% have a routine water intake of less than 2 L/day. An aberrant statistically significant negative correlation between total sleeping hours and mood (τb = -.313, p = 0.004) was identified, showing a negative effect on mood with a sleeping time of ≥ 9 hours when compared with the other two groups (6-9 hours, τb = - .689, p = 0.001, ≤ 6hours, τb = - .697, p = 0.001). A significant correlation between daily water intake and concentration was found, wherein a decrease in daily water intake showed a negative effect on concentration (τb = .289, p = 0.008).

**Conclusions:**

Sleep duration and water intake patterns may affect mood and cognitive performance. Regular sufficient nighttime sleep and adequate hydration may help improve cognitive functioning and mood.

## INTRODUCTION

Health encompasses both physical and mental well-being, with cognitive functioning and emotional stability playing crucial roles. Physical factors such as sleep and hydration significantly impact mental wellness. Sleep hygiene, which involves modifying behaviors related to sleep, like screen use and night-time food and beverage consumption, can enhance sleep quality. Adequate sleep in healthy adults is associated with improved cognitive functions, such as learning, concentration, and memory consolidation, while sleep deprivation leads to fatigue, declined cognitive performance, and mood changes.^1^

The relationship between sleep and cognitive functions is explained by synaptic connections functional during awake times, which are strengthened through sleep, leading to memory consolidation.^2^ Proper hydration is another vital determinant of optimal cognitive functioning. Water constitutes a significant portion of our body weight, and maintaining a balance between intake and output is crucial for hydration. Daily water intake recommendations are around two liters for females and 2.5 liters for males.^3^ Both excessive and inadequate water intake can negatively affect physical and cognitive performance.^4^

Considering the fast-paced urban lifestyle, it becomes imperative to understand the repercussions of lifestyle behaviour patterns on the mental well-being of individuals.^5^ We chose to include working adults, as well as college and university students, considering their inclination to prioritize various aspects of their lives over health, given their demanding schedules.^6^ This study aimed to identify the behaviour patterns of working adults regarding sleep and water intake in an urban environment and to find the association of sleep duration and water intake with mood, concentration and memory.

## METHODS

This was a descriptive correlational study conducted at Islamic International Medical College, Riphah University at the Pathology department from March to June 2022. A survey was conducted using a self-reporting questionnaire.Sample size was calculated using G*Power online calculator. With an effect size (r) of 0.3, a significance level (α) of 0.05, and a power (1- β) of 0.95, using the correlation test, the calculated sample size was 133 participants.

### Ethical Approval

It was taken from IRC Riphah International University (App.#Riphah/IRC/22/2031 dated 14^th^ February 2022).

### Inclusion criteria

for the participants was adults between 20 and 50 years of age, engaged in work or study commitments in the morning time. This inclusion was based on their routine to wake up early for these commitments, could expose them to potential effects of sleep deprivation. Individuals not committed to work or study in morning time, and those with any known depression or sleep disorder were excluded from participation.

The tool used for data collection was a comprehensive survey questionnaire titled “Lifestyle patterns and their effects”, developed by the authors in consultation with a lifestyle medical expert and validated by two lifestyle medicine and medical education experts. The questionnaire consisted of 44 questions in total, seven of them for demographic information. Twenty questions were related to the study objectives and used for data analysis. The questions were close ended and contained categorical data. It was developed on Google forms and piloted on a small sample (n=10) respondents to check for understanding and ambiguities. It was then distributed through email, Facebook and WhatsApp. Snowballing technique was also used to reach the desired population. A total of 200 forms were received, out of which 160 (80%) fulfilled the inclusion criteria. The questionnaire included demographic information and questions regarding sleep patterns, water intake, factors affecting sleep quality and hydration and perception of usual mood, concentration, and memory. Most of the questions were close-ended.

Data was analyzed using SPSS version 22. Descriptive data was calculated to assess the demographic data, sleep hours, daily water intake and other behavioral factors associated with sleep and water consumption. Relation of sleep duration and water intake with mood, mindfulness/concentration, and memory were determined using Kendall’s Tau-b correlation coefficient for different categorical variables as the data appeared to follow a monotonic relationship. A p-value of <0.05 was taken as statistically significant.

## RESULTS

A total of 200 forms were received, out of which 160 (80%) fulfilled the inclusion criteria. The demographic characteristics of the study subjects showed that 65% were females and 35 % were male participants. About 84% of the participants were between 20-30 years of age, 14% between 31-40 years, and 2% above 40 years. The percentage of students was 71%, whereas working adults were 29%. About 61 % of participants were engaged in study or work for 08 hours, 20% of the participants for less than eight hours and 19% for more than eight hours/day of study or work. Behaviour patterns of the participants regarding sleep duration and other factors relevant to sleep hygiene practices are given in (Table-I). When asked about the perceived mood, concentration and memory of relative lack of sleep, the participant’s response is given in (Table-II).

**Table-I:** Behavior patterns of adults regarding sleep and factors effecting sleep quality (n=160).

Parameters	Duration/Perception	Frequency	Percent
Average sleep hours	> 9 hours	16	10.0
	7-9 hours	98	61.3
	≤ 6 hours	46	28.7
Usual Dinner time	before 7pm	4	2.5
	7- 9 pm	68	42.5
	9-11 pm	76	47.5
	After 11pm	6	3.8
	Skip Dinner	6	3.8
Usual sleep time	8-10pm	30	18.8
	10pm to 12am	64	40
	After 12	66	41.3
Relation of meal intake with sleep time	Within 1 hour	16	10
	Between 1-2 hours	88	48.8
	> 2 hours	66	41.2
When is the screen usually used before sleeping	Switch off-screen just before sleep	132	82.5
	One hour before sleep	16	10
	Two hours before sleep	12	7.5
Usual wakeup time	4-6am	38	23.8
	6-8am	84	52.5
	after 8am	38	23.8
Working hours	< 8 hours	32	20
	8 hours	98	61.3
	>8 hours	30	18.8
Perceived workplace stress level	Comfortable	38	23.8
	Neutral	70	43.8
	Stressed	52	32.5

**Table-II:** Perceived effect of relative lack of sleep on mood, concentration and memory.

Parameters	Perception	Frequency	percent
Perceived effect of relative lack of sleep on mood	No effect	46	28.7
	Negatively affected	114	71.3
Perceived effect of relative lack of sleep on concentration	No effect	22	13.8
	Negatively affected	138	86.3
Perceived effect of relative lack of sleep on memory	No effect	46	28.7
	Negatively affected	114	71.3

Behavior patterns of participants regarding water intake showed that 63.7 % (n=102) participants had routine water intake of less than 2 L/day, 23.8% (n=38) two L/day and 12.6% (n=20) more than two L / day. 81.3% (n=130) participants reported drinking water at room temperature, 7.5% (n=12) took chilled water, and 11.3% (n=18) consumed mixed cold water. Regarding association of water intake with meals; 52.5 % (n= 100) individuals reported drinking water during meals, 25% (n=40) 30 minutes or more before meals, and 22.5% (n=38) about two hours after meals.

The correlation of usually perceived mood, concentration, and memory among subjects with different sleep and water intake patterns was calculated as given in (Table-III). A statistically significant negative correlation was identified between total sleeping hours and mood (τb = -.313, p = 0.004). When we compared between different groups, there was a significant adverse effect on mood with the sleeping time of ≥ 9hrs when compared with the other two groups (6-9 hours, τb = - .689, p = 0.001, ≤ 6 hours, τb = - .697, p = 0.001). A weak positive correlation of total sleeping hours with both concentration and memory was seen, but it was statistically insignificant (τb= .125, *p* = 0.249 and *p* = 0.362, τb= .071 *p* = 0.512 respectively).

**Table-III:** Correlation of mood, concentration, and memory with average sleep hours and daily water intake.

Parameters	Patterns of sleep/water intake	Mood % (n=)		Concentration % (n=)		Memory % (n=)		Kendall’s tau b correlation coefficient	(p-Value)

		Normal	Irritable	Normal	Takes some effort	Normal	Poor		
Sleep	>9 hours 10.0 (16)	1.5 (2)	50.0 (14)	0.0 (0)	19.5 (16)	8.6 (12)	18.2 (4)	-.313^a^	0.004^a^
	7-9 hours 61.3 (98)	68.2 (90)	28.6 (8)	71.8 (56)	51.2 (42)	65.2 (90)	36.4 (8)	.125^b^	0.249^b^
	≤ 6 hours 28.7 (23)	30.3 (40)	21.4 (6)	28.2 (22)	29.3 (24)	26.2 (36)	45.5 (10)	.071^c^	0.512^c^
Water intake	> 2L/day 12.5 (20)	10.6 (14)	21.4 (6)	17.9 (14)	7.3 (6)	11.5 (16)	18.2 (4)	-.025^d^	0.818^d^
	2L/day 23.8 (38)	25.8 (34)	14.3 (4)	33.3 (26)	14.6 (12)	24.6 (34)	18.2 (4)	.289^e^	0.008^e^
	< 2L/day 63.7 (102)	63.6 (84)	64.39 (18)	48.7 (38)	78 (64)	63.7 (88)	63.6 (14)	-.017^f^	0.876^f^

'a: sleep patterns with mood; 'b: sleep patterns with concentration; 'c: sleep patterns with memory, 'd: water intake with mood; 'e: water intake with concentration; 'f: water intake with memory, p value < 0.05 is significant.

A statistically significant positive correlation between daily water intake and concentration was found (τb = .289, p = 0.008), wherein a decrease in daily water intake negatively affected mood and memory. Still, it was feeble and statistically insignificant (τb= -.025, *p* = 0.818 & τb= -.017, *p* = 0.876 respectively) (Table-III).

## DISCUSSION

The purpose of the survey was to examine the sleep and water intake behavior of working adults in an urban setting and to determine if there was a connection between sleep duration, water intake, mood, and cognitive performance. Adults between 20 and 50 years of age, engaged in work or study commitments in the morning time were included in the study because of their need to wake up in time for their commitments in the morning, and their behaviour regarding sleep timing and sleep hygiene practices could potentially subject them to effects of sleep deprivation. Adults more than 50 years of age were excluded, as studies suggest changes in sleep patterns independent of work commitments with increasing age.^5^

Findings from the study showed that 61% (n= 98) participants reported sleeping for 7 to 9 hours, while a significant portion had sleep durations of less than six hours or more than nine hours (28.7% & 10% respectively). A considerable number of participants went to bed after midnight (41.3%), and around 41% slept between 10 PM and midnight. The wake-up times of participants varied, with the majority waking up between 6-8 AM (52 .5 %), and others waking up at 4-6 AM 23.8 %. The recommended sleep duration for young adults is 7-8 hours^7^, however shorter sleep spans have become more common among adults due to factors such as a 24-hours work culture and increased screen usage.^8^,^9^

Many workers and students have to wake up earlier to meet their job or study commitments, leading to a direct decrease in their sleep time. Sufficient sleep has lately been emphasized as a public health primacy in the Healthy People 2020 initiative, with both inadequate and prolonged sleep linked to increased probability of depression and poor health.^10^,^11^ A sleep span of < 7 hours is associated with an increased hazard of cardiovascular and cerebrovascular diseases, malignant neoplasms, accidents, septicemia and metabolic disorders.^12^ The quality and extent of sleep is also affected by workplace environment and job demands, with various studies linking workplace stresses to sleep disorders.^12^ Chronic sleep deprivation disrupts the body’s natural circadian rhythm, leading to reduced daytime alertness and energy. Also, poor sleep quality can contribute to depression and suicidal thoughts.^13^

Certain practices around bedtime that have crept in as modern social norms can negatively impact sleep duration and quality, which may be addressed through ‘sleep hygiene’.^14^ Limited research is available on the effects of late-night device usage, smoking, snacking, exercise, and alcohol consumption on sleep.^15^ The widespread use of electronic devices in bedrooms is associated with insufficient sleep in many adults. Higher screen media use is linked to lower sleep quality and quantity globally.^16^ In our study, 82.5% of participants reported using screens before sleep, which can delay sleep and affect its quality. Two factors contributing to poor sleep quality are the blue light emitted by devices and the radiofrequency electromagnetic fields (RF-EMFs) they emit, which stimulate the brain and disrupt melatonin production, making it harder to fall asleep.^17^

Observational studies have found a connection between poor sleep quality and consuming food close to bedtime.^18^ Recommendations are to have dinner one to two hours before sleep for better sleep. However, dinner timing can vary due to work and lifestyle constraints. In our study, a significant portion of participants reported having late dinners between 9-11 pm (47.5%), with 48.8% eating within two hours of sleep time and 10% within one hour. Late-night meals are generally advised against in sleep hygiene practices.

The literature suggests that food metabolism follows a circadian rhythm, and night-time food absorption is less efficient than during the day.^19^ Eating close to bedtime can lead to issues like heartburn, reflux, and abdominal discomfort. Relative sleep deprivation, has negative effects on learning, memory, brain function, cognitive processes, and emotional well-being. It can lead to mood changes, including irritability, which can affect one’s ability to handle daily pressures and strain relationships with colleagues.^16^

Our study found a significant negative correlation between sleep duration of nine hours or more and negative mood, supporting the findings from other studies.^20^ Water is essential for brain health, as it constitutes about 75% of the brain’s mass. Adequate hydration has been shown to improve mood and cognition in both children and adults.^21^ In our study, we examined water intake as a measure of hydration and found variations in daily water intake, with a significant number of participants consuming less than the recommended amount. While moderate variations in water consumption may not affect cognitive functioning in sedentary and temperate conditions, intense situations such as high temperatures, strenuous exercise, or military settings may impact these parameters.^22^ We observed a significant positive correlation between daily water intake and concentration (τb = .289, p = 0.008).

Although a weak negative correlation was found between decreased water intake and mood and memory, these associations were not statistically significant. Dehydration has been shown to impair cognitive functioning and mood in various studies.^23^ In our study, we found a significant correlation between daily water intake and concentration, indicating a negative impact of decreased water intake on concentration. Other research has also revealed that dehydration can affect cognitive functions, especially attention span and short-term memory.^24^ Some other studies found alterations in mood with inadequate hydration by fluid restriction alone, but cognitive functions were not affected.^25^

### Limitations

Firstly, it relied on survey responses, which are subjective and may be influenced by individual perceptions. Additionally, we used water intake as a simple indicator of hydration, without conducting more objective water deprivation tests.

## CONCLUSION

Our study concludes that a substantial portion of working adults in urban settings have suboptimal sleep duration and inadequate water intake patterns. We observed a statistically significant positive relation between the amount of water consumed daily and the level of concentration. We also noted a significant negative impact on mood when sleep duration exceeded more than eight hours. These findings highlight the intricate relationship between sleep, water intake, and mental well-being, emphasizing the need for balanced practices.

### Recommendations

Future studies should consider incorporating more rigorous and objective measures to assess the impact of hydration on the parameters discussed.

### Authors` Contribution:

**SQ:** Conceived, designed and did statistical analysis & editing.

**SI:** Did data collection and manuscript writing.

**MS & MA:** Did data collection, review and final approval of manuscript.
